# Genomic features of a multidrug-resistant and mercury-tolerant environmental *Escherichia coli* recovered after a mining dam disaster in South America

**DOI:** 10.1016/j.scitotenv.2022.153590

**Published:** 2022-06-01

**Authors:** Natália C. Gaeta, Daniel U. de Carvalho, Herrison Fontana, Elder Sano, Quézia Moura, Bruna Fuga, Patricio Montecinos Munoz, Lilian Gregory, Nilton Lincopan

**Affiliations:** a Department of Internal Medicine, School of Veterinary Medicine and Animal Science, University of São Paulo, São Paulo, Brazil; b Department of Preventive Veterinary Medicine and Animal Health, School of Veterinary Medicine and Animal Science, University of São Paulo, São Paulo, Brazil; c Department of Clinical Analysis, School of Pharmacy, University of São Paulo, São Paulo, Brazil; d One Health Brazilian Resistance Project (OneBR), Brazil; e Department of Microbiology, Institute of Biomedical Sciences, University of São Paulo, São Paulo, Brazil; f Federal Institute of Education, Science and Technology of Espírito Santo, Vila Velha, Brazil; g Institute of Geociences, University of São Paulo, São Paulo, Brazil

**Keywords:** Environmental pollution, Antimicrobial resistance, Genomic surveillance, Extended-spectrum β-lactamase, Mercury resistance, Critical pathogens, One health

## Abstract

Mining dam disasters contribute to the contamination of aquatic environments, impacting associated ecosystems and wildlife. A multidrug-resistant *Escherichia coli strain (*B2C) was isolated from a river water sample in Brazil after the Mariana mining dam disaster. The genome was sequenced using the Illumina MiSeq platform, and de novo assembled using Unicycler. Resistome, virulome, and plasmidome were predicted using bioinformatics tools. Data analysis revealed that *E. coli* B2C belonged to sequence type ST219 and phylogroup E. Strikingly, a broad resistome (antibiotics, hazardous heavy metals, and biocides) was predicted, including the presence of the clinically relevant *bla*_CTX-M-2_ extended-spectrum β-lactamase (ESBL) gene, *qacE∆1* efflux pump gene, and the *mer* (mercury resistance) operon. SNP-based analysis revealed that environmental *E. coli* B2C was clustered along to ESBL-negative *E. coli* strains of ST219 isolated between 1980 and 2021 from livestock in the United States of America. Acquisition of clinically relevant genes by ST219 seems to be a recent genetic event related to anthropogenic activities, where polluted water environments may contribute to its dissemination at the human-animal-environment interface. In addition, the presence of genes conferring resistance to heavy metals could be related to environmental pollution from mining activities. Antimicrobial resistance genes could be essential biomarkers of environmental exposure to human and mining pollution.

## Highlights

• Genomic data of a MDR *E. coli* recovered after a mining dam disaster are presented.
• A broad resistome (antibiotics, hazardous heavy metals, and biocides) was predicted.
• *E. coli* sequence type ST219 carried the clinically relevant *bla*_CTX-M-2_ ESBL gene.
• The presence of the *mer* operon conferring mercury tolerance is highlighted.
• The role of impacted rivers in the spread of critical priority pathogens is discussed.

## Graphical abstract

**Figure:**
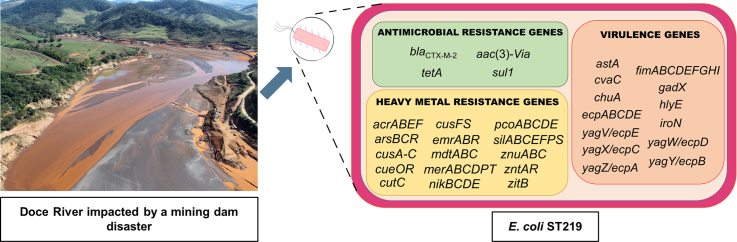

## 1 Introduction

Anthropogenic activities related to urbanization, industrialization, farming and animal food production have been responsible for introducing multidrug-resistant pathogens to aquatic environments in Latin America and worldwide (Dominguez et al., 2021). In this regard, although the World Health Organization (WHO) has deemed broad-spectrum cephalosporin-resistant and carbapenem-resistant Enterobacterales as critical-priority pathogens (Tacconelli et al., 2018), the magnitude of the threat of antimicrobial-resistance from polluted aquatic environments has not been quantified. While production of extended-spectrum β-lactamases (ESBLs) has been the main mechanism for broad-spectrum cephalosporins resistance in *Escherichia coli*, one of the well-known determinants of heavy metal resistance is the *mer* system (mercury resistant operon), which consists of a mercury reductase, a lyase, and other proteins (periplasmic, inner membrane, and regulatory) (Boyd and Barkay, 2012). Strikingly, heavy metals allow co-selection of antibiotic resistance due to co-resistance and cross-resistance mechanisms (Baker-Austin et al., 2006). In fact, heavy metal-resistant *E. coli* producing ESBLs have been reported abroad (Azam et al., 2018; Sütterlin et al., 2014; Freitas et al., 2019; Yang et al., 2020).

Dam rupture constitutes a disruption of nature-mediated mass transfer between geological reservoirs on Earth, inducing severe environmental damage. Specifically, anomalous enrichment of trace metals found in aquatic environments evidence this damage (Buch et al., 2020), contributing to the selection of environmental microbiota expressing tolerance to hazardous heavy metals (Keim et al., 2021). In addition, contamination by dam disaster tailings has increased the amount and abundance of antimicrobial resistance genes in the environment (Furlan et al., 2020).

In 2015, Brazil experienced an environmental accident after an iron ore dam failure in the Doce River basin, Minas Gerais state (Carmo et al., 2017), which raised the local environmental contamination and the bioavailability of heavy metals (Aguiar et al., 2020). Although, the presence of antibiotic resistance genes in environments contaminated with heavy metal, as well as the role of heavy metals in co-selection and horizontal transfer of plasmid-mediated antibiotic resistance genes have been well documented (Yang et al., 2017; Chen et al., 2019; Imran et al., 2019; Zhang et al., 2018), there is a lack of data about selection of critical priority pathogens and/or presence of resistant determinants in long-term metal contaminated areas affected by environmental accidents. In this study, we have conducted a genomic and microbiologic investigation of WHO critical priority pathogens recovered from water samples collected from a river affected by a mining dam disaster.

## 2 Materials and methods

A local surveillance study was conducted to investigate the occurrence of critical priority bacteria along 84 km of the Doce River Basin (Minas Gerais State, southeastern Brazil), affected by a mining dam disaster. During 2018, samples were obtained from eight different sites comprising urban and rural areas. Briefly, 500 mL of surface water of each site were collected using sterile plastic bottles, and they were stored and transported to the laboratory at 4 °C. Samples were concentrated by filtering 100 mL of each sample using a 0.45 μm sterile membrane. To ensure maximum bacterial isolates, another 100 mL of water samples were centrifugated (5000 rpm/30 min). Filters and pellets were suspended in three milliliters of Brain Heart Infusion broth (Difco, USA) and incubated at 35 °C ± 2 °C for 24 h. Growths were used in the following steps.

### 2.1 Bacterial isolation, species identification and antibiotic susceptibility profile

Ten microliters of each broth with bacterial growth were streaked on MacConkey agar plates (Acumedia) supplemented with ceftriaxone (2.0 μg/mL). Bacterial strains were identified by matrix-assisted laser desorption ionization-time of flight mass spectrometry (MALDI-TOF MS). The antimicrobial susceptibility profile was determined by Kirby-Bauer method (CLSI, 2021), and ESBL production was screened using the double-disk synergy test (Rawat and Nair, 2010; CLSI, 2021).

### 2.2 Heavy metal tolerance

Tolerance to sodium arsenite (AsNaO_7_; Baker & Adamson, USA), mercury chloride (HgCl_2_; Mallinckrodt, UK), copper sulfate (CuSO_4_.5H_2_O; Synth, Brazil), silver nitrate (AgNO_3_; Merck, Germany), cobalt chloride (CoCl_2_; Baker & Adamson, USA), and potassium dichromate (K_2_Cr_2_O_7_; Vetec, Brazil) was evaluated using a broth microdilution method (CLSI, 2021). *Klebsiella pneumoniae* strain KPN535 (One260, http://www.onehealthbr.com/), harboring mercury (*merA-C*), arsenic (*arsA-D*, *arsH*), copper (*pcoA-E*, *pcoR-S*), silver (*silR-S*, *silC*, *silE*), and nickel (*nikA-E*) resistance genes, and the *E. coli* ATCC strain 25922, negative for the presence of genes conferring tolerance to heavy metal tested, were used as controls.

### 2.3 Genomic analysis

DNA extraction was performed using the PureLink® Genomic DNA Mini Kit (Thermo Fisher Scientific, USA) following the manufacturer's instructions. DNA concentration was assessed by Qubit® 2.0 fluorometer (Life Technologies, Carlsbad, CA). The genomic library was constructed using the Nextera XT DNA Library Preparation Kit (Illumina Inc., Cambridge, UK). The genome of the *E. coli* B2C strain was sequenced on an Illumina MiSeq platform (Illumina Inc., San Diego, CA) using 2 × 150 bp paired-end reads. Adaptors were removed using Trim Galore v.0.6.5 (https://github.com/FelixKrueger/TrimGalore). Then, filtering and trimming were performed using AfterQC v.0.9.7 (https://github.com/OpenGene/AfterQC) and Trimmomatic v.0.36, with the following parameters: LEADING = 20, TRAILING = 20, SLIDINGWINDOW = 4:20, HEADCROP = 10, CROP = 235, and MINLEN = 85 (Bolger et al., 2014). Filtered and trimmed reads were de novo assembled using Unicycler v.1.2.10 (https://github.com/rrwick/Unicycler) (Wick et al., 2017). The draft genome sequence was automatically annotated using Rast: Rapid Annotation Using Subsystem Technology v. 2.0 server (https://rast.nmpdr.org) (Aziz et al., 2008) and Prokka (https://github.com/tseemann/prokka) (Seemann, 2014). Genome size was calculated using Jellyfish v.2.3.0 (https://github.com/gmarcais/Jellyfish). Antimicrobial resistance genes were assessed using ResFinder v.4.0 (Zankari et al., 2012), Megares 2.0 (Doster et al., 2019), and Bacmet v.2.0 (https://bacmet.biomedicine.gu.se) databases. Virulome, plasmidome, multilocus sequence typing (MLST), plasmid multilocus sequence typing (pMLST), fimH-type, and serotype were predicted using VFDB (Chen et al., 2016) and Ecoli_vf (https://github.com/phac-nml/ecoli_vf), PlasmidFinder (Carattoli et al., 2014), MLST 2.0.4, pMLST 0.1.0, FimTyper 1.0, and SeroTypeFinder 2.0.1, respectively, available at the Center for Genomic Epidemiology (https://genomicepidemiology.org/). Prediction of contigs as plasmid-derived was performed using mlplasmids v.3.4.1tool (https://gitlab.com/sirarredondo/mlplasmids) (Arredondo-Alonso et al., 2018). Phylogroup was determined in silico using the ClermonTyping tool (http://clermontyping.iame-research.center/) (Beghain et al., 2018). A ≥ 95% identity threshold was used to identify all genes.

### 2.4 Phylogenetic analysis

Raw reads of *E. coli* were submitted to the *Escherichia*/*Shigella* database in Enterobase (https://enterobase.warwick.ac.uk). The genome assembly of *E. coli* strain B2C (Enterobase Uberstrain: ESC_WA0085AA) was downloaded from Enterobase, along with all available genome assemblies from ST219 lineages, which had data for source of isolation, country, and year of collection. A total of 34 *E. coli* ST219 assemblies were downloaded. The B2C genome was compared to each genome of the same ST to assess the average nucleotide identity (ANI), using FastANI v1.32 (https://github.com/ParBLiSS/FastANI). All downloaded genomes of *E. coli* ST219 were used with B2C for phylogenetic tree construction. CSI Phylogeny v1.4 (https://cge.cbs.dtu.dk/services/CSIPhylogeny) was used with default settings to generate a SNP-based maximum-likelihood phylogenetic tree and a distance matrix with SNP counts. Chromosome sequence of *E. coli* ST219 strain EC974 (RefSeq accession number: NZ_CP021840.1) was used as reference. iTOL v6 (https://itol.embl.de) was then used for midpoint-rooting and annotating the tree with data from Enterobase and ABRicate.

## 3 Results and discussion

**Fig. 1:**
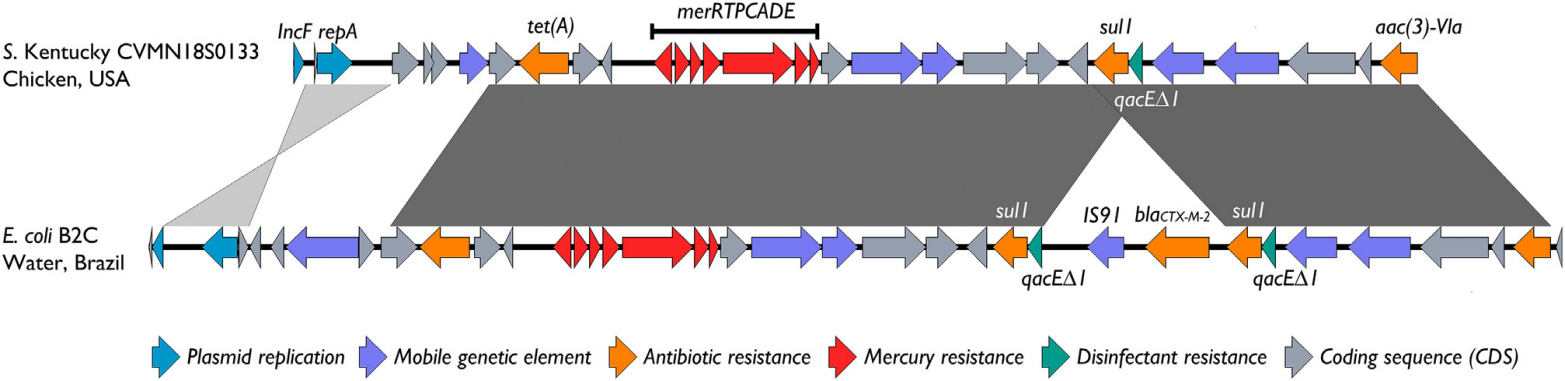
Schematic representation *E. coli* B2C IncFIB plasmid (this study), and *Salmonella* Kentucky plasmid isolated from chicken wing in USA (Genbank accession no. CP082700). The *bla*_CTX-M-2_ gene is associated with *IS*91 insertion sequence, along with the mercury resistance operon (*merETPCADE*), antibiotic [*sul1*, *tet(A)*, *aac(3)-Vla*] and quaternary ammonium compounds (*qacEΔ1*). Arrows represents coding sequences (CDS) labeled with gene name or product and displayed according to gene orientation; the gray shades indicates regions of homology.

A broad-spectrum cephalosporin-resistant *E. coli* (strain B2C) was recovered from the Doce River Basin (20°17′35.0″S; 43°11′36.6″W). This strain showed a multidrug-resistant profile to ceftriaxone, cefotaxime, cefepime, sulfamethoxazole/trimethoprim, amikacin, gentamicin, and tetracycline; remaining susceptible to aztreonam, nalidixic acid, ciprofloxacin, ceftazidime, cefoxitin, ertapenem, imipenem, and meropenem. No intermediate resistance was found. Indeed, resistome analysis revealed genes encoding resistance to β-lactams (*bla*_CTX-M-2_), sulphonamide (*sul1)*, tetracycline (*tetA*), and aminoglycosides [*aac*(3)-*Vla*] (Table 1). In this regard, the *bla*_CTX-M-2_ ESBL gene has been relevant in South America (Bevan et al., 2017). The *bla*_CTX-M-2_ ESBL gene in the B2C strain was next to the *qacE∆1* gene associated with disinfectant resistance (Tong et al., 2021), being flanked upstream by an IS*91*-type insertion sequence element (Fig. 1). In this regard, disinfectants and heavy metals are co-selecting substances that contribute to the spread of antibiotic resistance genes (Baker-Austin et al., 2006; Pal et al., 2015).

**Table 1:** Genomic and epidemiological data of environmental *Escherichia coli* strain B2C.

Characteristics	*E. coli* B2C
Source	Aquatic environment
ATB resistance profile^a^	CRO, CTX, CPM, AMI, GEN, TET, SXT
Genome Size (bp)	5,391,093
No. of CDSs^b^	4618
G + C content (%)	50.62
tRNAs (*n*)	82
rRNAs (*n*)	6
MLST (ST)^c^	ST219
wgMLST	195,094
cgMLST	171,825
rMLST	1777
Serogroup	O:H16
Phylogroup	E
FimH-type	370
Resistome	
β-lactams	*bla*_CTX-M-2_
Aminoglycosides	*aac(3)-*Via
Tetracyclines	*tet*A
Sulfonamides	*sul1*
Heavy metals^d^	*arsBCR*, *cueOR*, *cusA-C*, *cusF*, *cusS*, *cutC*, *emrA*, *emrB*, *emrR*, *mdtA*, *mdtB*, *mdtC*, *merA*, *merC*, *merD*, *merT*, *merP*, *merR*, *nikB*, *nikC*, *nikD*, *nikE*, *pcoA*, *pcoB*, *pcoC*, *pcoD*, *pcoE*, *silA*, *silB*, *silC*, *silE*, *silF*, *silP*, *silS*, *znuA*, *znuB*, *znuC*, *zntA*, *zntR*, *zitB*, *zraR*
Disinfectant^e^	*qacE∆1*, *acrA*, *acrB*, *acrE*, *acrF*, *acrS*
Virulome	*astA, cvaC, chuA, ecpA, ecpB, ecpC, ecpD, ecpE, fimA, fimB, fimC, fimD, fimE, fimF, fimG, fimH, fimI, gadX, hlyE, iroN, yagV/ecpE, yagW/ecpD, yagX/ecpC, yagY/ecpB, yazZ/ecpA*
Plasmidome	IncFII, IncFIB
pMLST	F24:A-:B1
GenBank accession number	JACSWD000000000.1

a ATB, antibiotic. CRO, ceftriaxone; CTX, cefotaxime; CPM, cefepime; AMI, amikacin; GEN, gentamicin; TET, tetracycline; SXT, sulfamethoxazole/trimethoprim.

b CDSs, coding sequences.

c MLST, multilocus sequence type; ST, sequence type.

d *ars* (arsenic, antimony), *cue* (copper), cus (copper, silver), *cut* (copper), *emr* [phenylmercury acetate, 2-chlorophenylhydrazine, carbonylcyanide *m*-chlorophenyl hydrazone (CCCP), tetrachlorosalicylanilide (TCS), carbonyl cyanide 3-chlorophenylhydrazone (CCCP)], *mdt* (zinc), *mer* (mercury), *nik* (nickel), *pco*, (copper), *sil* (silver), *znu* (zinc), *znt* (lead, cadmium, zinc), *zit* (zinc), *zra* (zinc) (http://bacmet.biomedicine.gu.se/).

e *qacE∆1* [benzylkoniumchloride (BAC), ethidium bromide, acriflavine, chlorhexidine, pyronin Y, rhodamine 6G, methyl viologen, tetraphenylphosphonium (TPP), 4,6-diamidino-2-phenylindole (DAPI), acridine orange, sodium dodecyl sulfate (SDS), sodium deoxycholate (SDC), crystal violet, cetrimide (CTM), cetylpyridiniumchloride (CPC), dequalinium], *acr* [acriflavine, sodium dodecyl sulfate (SDS), sodium deoxycholate (SDC), tetraphenylphosphonium (TPP), benzylkoniumchloride (BAC), methyl viologen, ethidium bromide] (http://bacmet.biomedicine.gu.se/).

For B2C strain, tolerance to mercury chloride (MIC, 8 μg/mL) was three times greater than for *E. coli* ATCC 25922 (MIC, 1 μg/mL), and identical (MIC, 8 μg/mL) to the one shown by the *mer* operon-positive *K. pneumoniae* KP535 strain (Table 2). In this respect, heavy metal resistance genes for mercury (*merACDTPR*), arsenic (*arsRBC*), nickel (*nikABCDE*), copper (*pcoABCDR*, *cusABCFRS*, *cueOR*, *cutA*), zinc (*znuABC*, *zntAR*, *zitB*, *zraR*), and silver (*silABCEFPS*) were identified in *E. coli* B2C strain. The presence of several heavy metal resistance genes in *E. coli* strain B2C seems unusual compared to other *E. coli* strains recovered from the environment (Azam et al., 2018; Siddiqui et al., 2020). However, this difference could be due to the use of whole-genome sequencing, whereas most studies describe the detection of a few selected genes using PCR alone. Therefore, our finding could give more evidence about the role of environmental areas contaminated with heavy metal in the selection of bacterial isolates acquiring antimicrobial resistance genes.

**Table 2:** Minimum inhibitory concentrations (μg/mL) of heavy metals for *E. coli* B2C, and *E. coli* ATCC 25922 and *K. pneumoniae* KPN535 control strains.

Heavy-metal	Minimum inhibitory concentration (μg/mL)		
	*E. coli* B2C	*E. coli* ATCC 25922	*K. pneumoniae* KPN535
Arsenic (AsNaO_7_)	512	32	> 1024
Cobalt (CoCl_2_)	1024	512	512
Copper (CuSO_4_.5H_2_O)	1024	1024	2048
Chromium (K_2_Cr_2_O_7_)	256	128	128
Mercury (HgCl_2_)	8	1	8
Silver (AgNO_3_)	2	4	4

**Fig. 2:**
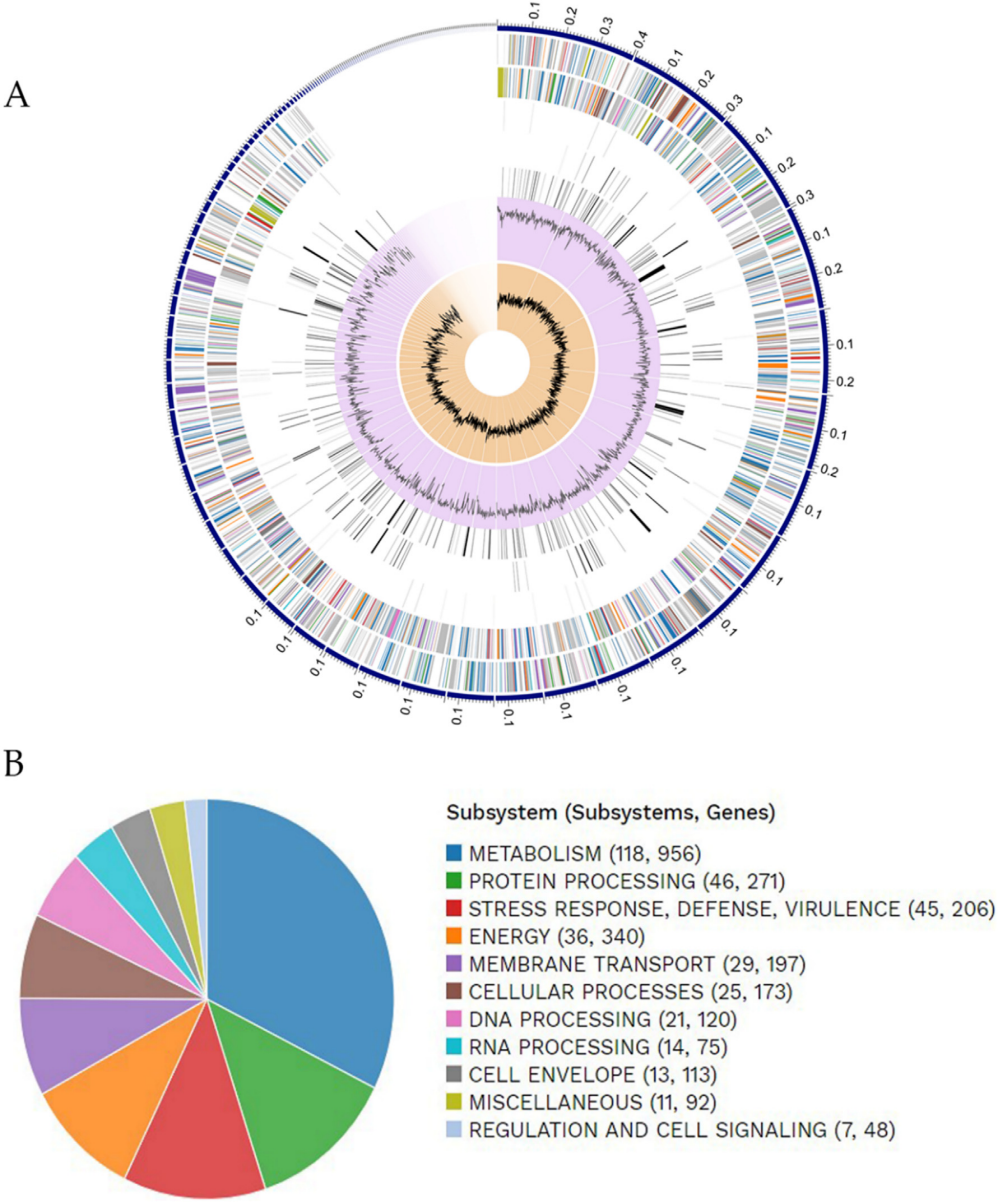
In A, a circular graphical plot of *Escherichia coli* strain B2C shows the genome annotation distribution. From outer to inner rings: the contigs, CDS (forward strand), CDS (reverse strand), RNA genes, CDS with homology to known antimicrobial resistance genes, CDS with homology to known virulence factors, GC content, and GC skew. In B, PATRIC functional annotation and overview of subsystems genes in the environmental *E. coli* strain B2C. The color scheme also indicates the subsystem that belongs to each CDS gene on the forward and reverse strand.

Whole-genome sequence analysis generated 2,290,538 paired-end reads assembled in 139 contigs, with 107× coverage, N_50_ value of 159,706, and a G + C content of 50.62%. The B2C strain presented a genome size of 5,391,093 bp, containing 82 tRNAs and six rRNAs. Circos plot (Zrywinski et al., 2009) and subsystem annotation obtained from the RAST server are shown in Fig. 2A and B, respectively.

Virulome analysis identified salmochelin (*iroN), colicin V (cvaC), type 1 fimbriae (fimH), outer membrane heme/hemoglobin receptor (chuA), hemolysin E (hlyE), heat-stable enterotoxin (astA), and E. coli common pilus (ecpA-E) genes.*

The *E. coli* B2C strain also harbored a plasmid of incompatibility group IncFIB (belonging to FAB formula F24:A-:B1), which has already been detected in *E. coli* strain harboring the *bla*_CTX-M-2_ gene from seagulls (*Laurus dominicanus*) in Argentina (Liakopoulos et al., 2016). The IncF F24:A-:B1 has also been referred to as a successful plasmid with wide dissemination among pathogenic or commensal *E. coli* strains (Liakopoulos et al., 2016).

Although, in this study, was not possible to obtain the complete nucleotide sequence of plasmids due to limitations of short-read methodology, analysis using mlplasmids v.2.1.0 (https://sarredondo.shinyapps.io/mlplasmids) showed that the ESBL-encoding *bla*_CTX-M-2_ gene and the mercury resistance operon (*merRTPCADE*) were carried by the IncF [F24:A-:B1] plasmid, along with genes encoding resistance to tetracycline (*tetA*), sulfonamides (*sul1*), aminoglycosides [*aac(3)-Vla*] and quaternary ammonium efflux pump (*qacEΔ1*). Additionally, the *bla*_CTX-M-2_ gene was found to be located upstream of an *IS*91 insertion sequence (Fig. 1), being both flanked by duplicated copies of *sul1-qacEΔ1* genes that may have resulted from an insertion sequence event (Poirel et al., 2008). Furthermore, blastn (https://blast.ncbi.nlm.nih.gov/Blast.cgi) analysis of the pB2C plasmid partial sequence (33.7 kb in length) showed that it was highly similar to an IncF [F16:A-:B-] plasmid identified in *Salmonella enterica* serovar Kentucky isolated from chicken wing in the United States of America (Genbank accession number CP082700), sharing 99.99% nucleotide pair-wise identity and 82% query coverage. This finding denote how these genes have been disseminated in aquatic environments, highlighting the possibility of gene transfer from/to other species (Chen et al., 2019; Fu et al., 2019).

The B2C strain was assigned to phylogroup E, commonly associated with bovine lineages (Coura et al., 2015a; Coura et al., 2015b). In this regard, the Doce river basin has dairy farms along its shore, and most cows have free access to the river, where feces can reach the watercourse contributing to the bacterial load.

*The E. coli* B2C strain was assigned to the ST219 clone, previously identified in companion animals in France (Melo et al., 2019), and human hosts in Tunisia (Ben Sallem et al., 2012). *Moreover, the average nucleotide identity (*ANI) of 33 genomes of *E. coli* ST219 that were compared to the B2C strain ranged between 99.4639% and 99.9561%. On the other hand, in the phylogenetic analysis, the percentage of reference genome covered by all isolates was 84.80%, corresponding to 4,382,433 positions found in all analyzed genomes. Single-nucleotide polymorphisms (SNPs) count among all 34 genomes of ST219 analyzed ranged between 0 and 5,273 SNPs, when using default settings (Minimum distance between SNPs of 10 bp) (Supplementary material 1). Disabling minimum distance between SNPs raised maximum SNP distance among the genomes to 10,163; but raising the distance to 100 bp reduced maximum SNP distance to 1,076. However, the resulting trees had similar clusters to the one generated with default settings. High differences in SNPs values highlight the degree of genetic variation among ST219 strains, with relatively distant strains within the same ST. However, the low number of available ST219 genomes on Enterobase may also interfere in this result. Finally, it is important to point out that contigs were used for the phylogenetic analysis instead of reads. Therefore, the minimum distance between SNPs (pruning) was the only possible adjustment in CSI Phylogeny because the other parameters are related to the quality of reads.

**Fig. 3:**
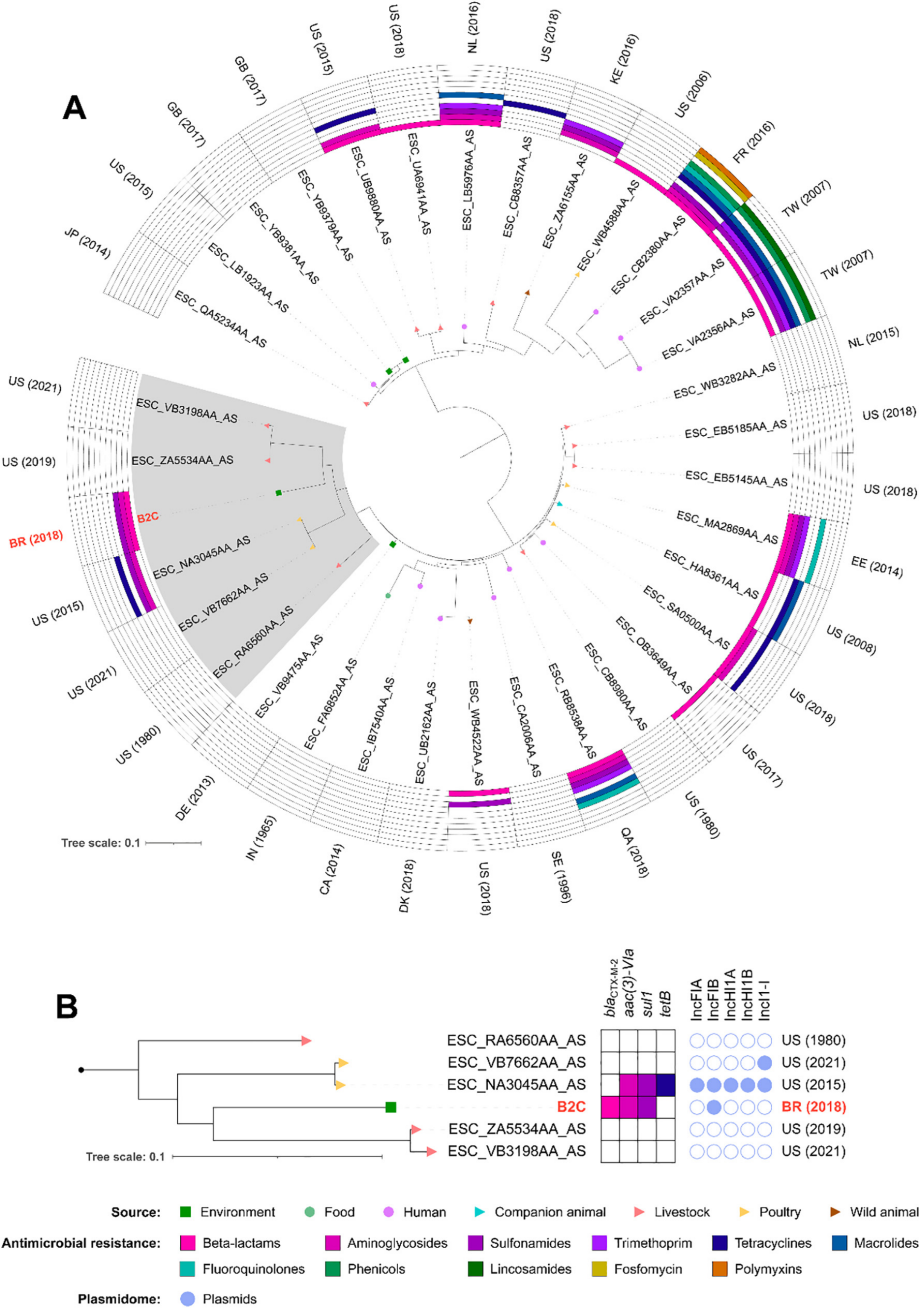
In A, phylogenetic tree of 34 *Escherichia coli* strains belonging to ST219. Genome sequences and epidemiological information (i.e., sources of isolation, predicted antimicrobial resistance phenotype, country, and year of collection) were downloaded from Enterobase (www.enterobase.warwick.ac.uk). ISO 3166-1 Alpha-2 country codes: BR, Brazil; CA, Canada; DE, Germany; DK, Denmark; EE, Estonia; FR, France; GB, United Kingdom; IN, India; JP, Japan; KE, Kenya; NL, Netherlands; QA, Qatar; SE, Sweden; TW, Taiwan; US, United States. In B, a subtree of the highlighted cluster shows the resistome and plasmidome of the isolates.

The phylogenetic tree clustered the B2C strain with five ESBL-negative *E. coli* ST219 strains isolated between 1980 and 2021, from livestock and poultry, in the United States of America (USA) (Fig. 3). Besides to the phylogroup E assignment, the phylogenetic clustering supports the hypothesis that *E. coli* B2C can be an animal (mostly livestock)-derived lineage.

Anthropogenic activities (e.g., industrial, household, hospital, and agricultural) are responsible for releasing large volumes of waste in the watercourses and contribute to the spread of antimicrobial-resistant bacteria (Squadrone, 2020). The presence of antibiotic- and heavy metal-resistant bacteria in aquatic environments can further contribute to the dissemination of antibiotic resistance genes to associated ecosystems (Gomi et al., 2017). In fact, a previous metagenome investigation on bacterial microbiota and resistomes in cows from dairy farms at the Doce River basin region revealed the presence of Enterobacterales and ESBL genes (Gaeta et al., 2020). Therefore, critical priority pathogens can achieve animal and human host throughout water and contaminated food crops (Verraes et al., 2013; Oniciuc et al., 2019).

A particular concern about heavy metals and antibiotic resistance is that metals are not biodegradable and involve long-term selective pressure (Song et al., 2017). Heavy metals are agents that contribute to the indirect selection of antibiotic resistance genes, mainly by co-selection of cross-resistance (Pal et al., 2018). The co-selection is essential to disseminate and maintain antibiotic resistance even in pristine environments, where no or few antibiotics are used (Pal et al., 2018). Cross-resistance occurs when a single system, such as an efflux pump, confers resistance to different determinants (e.g., disinfectants, antibiotics, and heavy metals) (Chapman, 2003; Baker-Austin et al., 2006). On the other hand, co-resistance occurs when distinct antimicrobial resistance genes are physically linked to the same genetic element (e.g., transposon, plasmid). Consequently, resistance to one antimicrobial compound results in simultaneous resistance to others (Baker-Austin et al., 2006).

The presence of different resistance genes within the same genetic context has a particular concern regarding horizontal gene transfer when a conjugative plasmid is involved (Rasmussen and Sorensen, 1998). In this regard, heavy metals seem to facilitate the conjugative transfer in environmental bacteria. Indeed, copper and zinc were found to accelerate the conjugative transfer of antimicrobial resistance genes in freshwater bacteria (Wang et al., 2020; Wang et al., 2021), whereas *E. coli* strains co-harboring antibiotic and heavy metal resistance (e.g., *mer* operon and/or *silEPS*, *merBPT*, and *arsC*) genes have been described in contaminated aquatic environments (Azam et al., 2018; Chen et al., 2019; Sultan et al., 2020)*.*

Our findings corroborate the genetic linkage between *mer* operon and antibiotic resistance genes in bacterial isolates found in polluted aquatic environments (McIntosh et al., 2008). Strikingly, in the *E. coli* B2C strain, the mercury resistance operon and antibiotic and disinfectant resistance genes were located on the same mobile genetic element (i.e., IncF plasmid). Thus, our results reinforced the evidence about the correlation between antibiotic resistance and mine-related contamination, and suggest the occurrence of both the co-resistance mechanism and horizontal gene transfer among bacteria in the Doce River basin.

Lately, the environmental co-selection of resistance to cephalosporins and tetracyclines by selective mercury pressure has been demonstrated in *Bacillus* spp. isolates from a mining district in Almadén, Spain (Robas et al., 2021). Thus, our results reinforced the evidence about the correlation between antibiotic resistance and mine-related contamination.

Dam rupture constitutes a severe disruption resulting in a severe and significant mass transfer between geological reservoirs on Earth, inducing environmental damage visible in microbiota, flora, fauna, soils, and water. For instance, we know that 60 Mt. of mining waste from the Mariana dam was released into Rio Doce ecosystems on November 5, 2015. For comparison, a transfer rate of 1.6 Gt/year of continental debris has been quantified for the West African region, which means ~43 Mt./day of matter transfer (Grimaud et al., 2018). A similar tectonic context shared by West Africa and Brazil supports that analogy. The massive quantity of iron tailings from Mariana dam implies a transfer of several trace metals (i.e., Al, As, Cd, Cr, Cu, Fe, Hg, Mn, Ni, Pb and Zn), leading to bioaccumulation of x3.2 times than those attained in a laboratory for Hg (Buch et al., 2020). The bioaccumulation of metals in the environment incontestably causes adverse effects on biological processes and may contribute to the selection and dissemination of antimicrobial resistance genes (Baker-Austin et al., 2006; Buch et al., 2020). Indeed, a significant correlation between heavy metal bioaccumulation in fish muscle and the occurrence of heavy metal resistance genes in *E. coli* has been described (Ture et al., 2020).

Consequently, iron tailings also imply an anomalous enrichment of trace metals becoming toxic for several species. In addition, a dramatic consequence is time-related, given that residence time for Hg is around 20 to 30 years in oceanic waters (Gworek et al., 2016). Therefore, it is highly advisable to monitor the consequences of this dam rupture on Brazilian marine waters.

Data on the background levels of antimicrobials in the Doce River basin's water and/or sediments are scarce. Approximately 60 Mt. of mining tailings were responsible for increasing the concentration of some metals. Copper, nickel, and zinc were higher in the reducible sediment fractions and associated with the tailings' original composition (Aguiar et al., 2020). A recent study comparing trace metals in sediments before and after the disaster showed that the mud was the source of cadmium, and arsenic was present before the environmental disaster. However, its concentration increased due to sediment remobilization (Duarte et al., 2021).

The Samarco Company refers that the mud did not contain a dangerous concentration of heavy metals, and it was composed mainly of silt (47.5%), followed by fine sand (37.5%), clay (10.6%), and coarse sand (4.5%). However, independent studies such as those conducted by the Minas Gerais State Water Agency (IGAM) detected high levels of mercury, arsenic, cadmium, copper, chromium, lead, zinc and nickel in water and/or sediments samples from the Doce River following the rupture (IGAM, 2022), suggesting that the ore tailings may also have minor and trace elements, as found in the Brumadinho slurry (Vergilio et al., 2020).

Copper, nickel, and aluminum were above the maximum permissible value by Brazilian legislation in the water **2** years after de accident (Carvalho et al., 2017). Indeed, results from a water and sediment quality assessment in the coastal zone around the mouth of Doce River after the accident indicated that the dam rupture affected water and sediment quality in the Atlantic Ocean but also showed that the concentrations of the toxic elements are returning slowly to the levels before the accident (Richard et al., 2020). Finally, water samples used in the present study were also evaluated by wavelength dispersive X-ray fluorescence (data not published). The mean concentration of aluminum (3.79 mg/L), copper (0.2 mg/L), and iron (15.4 mg/L) were above the Brazilian regulations (CONAMA. National Environment Council, 2005), while mercury, cobalt, and arsenic were not detected. Therefore, even though the long history of pollution of the Doce River basin (domestic and mining effluents containing toxic elements), the mining tailings spill may have potentiated the heavy metal contamination in the river (Richard et al., 2020; Santana et al., 2021), and increased the selective pressure on bacteria, regarding antimicrobial resistance genes.

In summary, we report genomic and microbiological data of an environmental *E. coli* belonging to ST219, co-harboring the clinically relevant *bla*_CTX-M-2_ ESBL gene and *mer* operon genes conferring tolerance to mercury (a hazardous waste problem); recovered from a Brazilian river impacted by a mining dam disaster. Our results suggest that the acquisition of clinically relevant resistance genes by the environmental *E. coli* ST219 seems to be a genetic event related to anthropogenic activities. In contrast, the presence of genes conferring resistance to heavy metals could be related to environmental pollution from mining activities. Therefore, antimicrobial and heavy metal resistance genes could be essential biomarkers of environmental exposure to human and mining pollution.

## Nucleotide sequence accession number

This Whole Genome Shotgun project has been deposited at DDBJ/ENA/GenBank under the accession JACSWD000000000. The version described in this paper is version JACSWD000000000.1 (Bio Project PRJNA658122).Genomic information of *E. coli* B2C strain is available on the OneBR platform under the number ID ONE113 (http://onehealthbr.com/).

The following is the supplementary data related to this article.

Supplementary material 1 SNP matrix ST219. Supplementary material 1

## Funding

This work was funded in part by the 10.13039/100000865Bill & Melinda Gates Foundation, United States [Grand Challenges Explorations Brazil – New approaches to characterize the global burden of antimicrobial resistance, Grant OPP1193112], 10.13039/501100001807Fundação de Amparo à Pesquisa do Estado de São Paulo (2020/08224-9), and 10.13039/501100003593Conselho Nacional de Desenvolvimento Científico e Tecnológico (AMR 443819/2018-1, 312249/2017-9 and 433128/2018-6). N.C. is a fellow of FAPESP (2016/23204-9). H.F. is a research fellow of CAPES (88887.506496/2020-00). B.F. is a research fellow of CAPES (88887.358057/2019-00). N.L. is a research fellow of CNPq (314336/2021-4). L.G. is a research fellow of CNPq (312249/2017-9).

## CRediT authorship contribution statement

**Natália C. Gaeta:** Conceptualization, Methodology, Investigation, Formal analysis, Writing – original draft. **Daniel U. de Carvalho:** Investigation. **Herrison Fontana:** Investigation, Formal analysis. **Quézia Moura:** Investigation. **Bruna Fuga:** Investigation. **Patricio Montecinos Munoz:** Writing – review & editing. **Lilian Gregory:** Conceptualization, Writing – review & editing, Supervision, Project administration, Funding acquisition. **Nilton Lincopan:** Conceptualization, Methodology, Resources, Writing – review & editing, Supervision.

## Declaration of competing interest

The authors declare no conflict of interest.
